# Design of an integrated model using U-Net, DeepSurv, and cross-attention for lung cancer classification and survival prediction

**DOI:** 10.1038/s41598-025-29781-x

**Published:** 2025-12-03

**Authors:** Mattakoyya Aharonu, LokeshKumar Ramasamy

**Affiliations:** School of Computer Science and Engineering, Vellore Institute of Technology, Vellore, Tamil Nadu 632014 India

**Keywords:** Lung cancer, U-Net, DeepSurv, Multimodal fusion, Survival prediction, Cancer, Computational biology and bioinformatics, Mathematics and computing

## Abstract

Lung cancer ranks within the highest mortality rates among cancerous diseases; hence, its detailed classification and survival rate prediction are of utmost importance. Most existing approaches for the classification and prognosis prediction in lung cancer share a critical deficiency: they are either single-modality or fail to learn complex, nonlinear interactions between distinct data types. However, none of these traditional models iteratively refines segmentation with the requisite accuracy to embed continuous flow of new patient data without degradation in performance. We hence propose an Iterative Multi-Model Deep Learning Framework for improved classification of lung cancer subtypes and predictions of survival rates. Our proposed work uses the U-Net model, which refines features extracted iteratively to improve precision in segmented regions. For lung cancer subtypes classification, feature-level fusion is done by using CNN for spatial features extracted from both radiological and histopathology images and using an MLP for genomic data samples. The DeepSurv model extends the Cox proportional hazards model with deep learning to handle complex, multi-dimensional clinical, imaging, and genomic data for survival rate prediction. Bayesian optimization is used to optimize the hyperparameter tuning process, whereas EWC empowers this approach with real-time survival predictions, thus enabling incremental learning without catastrophic forgetting. This is further reinforced by a multimodal attention mechanism that ensures the most discriminative features from each modality are taken into consideration by the model. The contributions of this work consist of an improvement in tumor segmentation accuracy with results that range from 90 to 95% Dice similarity, a raise of accuracy in lung cancer subtype classification between 85% and 90%, and robust survival rate predictions with a C Index of ~ 0.75–0.80. Besides, our adaptive learning approach can continuously improve our model to make it fit for real-time clinical applications. The framework will present an end-to-end solution for the diagnosis and prognosis of lung cancer.

## Introduction

 Lung cancer is one of the most common and deadliest cancers around the world, responsible for a high percentage of cancer deaths. Early diagnosis, together with the effective prognosis of the patient’s survival, is considered to play a very critical role in improving outcomes. However, lung cancer is greatly heterogeneous; there exist several subtypes which differ essentially from one another in genetics, histology, and radiology. This is a complex disease, and existing diagnostic and prognostic models have considerable limitations in managing the data modalities and making appropriate predictions. Conventionally, lung cancer diagnosis is done via imaging, usually through CT images, on which radiologists can identify and segment lung lesions. While segmentation plays an important role in diagnosis, the task largely depends on the expertise of the radiologist and hence is subject to interobserver variability sets. Recently, CNNs have emerged as a strong tool for image segmentation and obtained much success in the area of medical image analysis. Among them, U-Net has emerged as one of the standard architectures in biomedical image segmentation since it can model both global and local features using an encoder-decoder structure with skip connections. Although U-Net has been very successful for the segmentation task, one major limitation with that approach is the fixed single-pass feature extraction process that may not fully capture complex features associated with lung tumors. This is possibly true, especially considering those scans prepared from different imaging modalities such as CT scans and histopathology images. Therefore, there is a requirement for iterative refinement of feature extraction that enables more precise and detailed delineation of lung tumor regions.

Complementary to imaging, there is an emerging need for genomic information about gene mutations and RNA-Seq data that will further elucidate the molecular underpinnings of lung cancer subtypes. Genomic data informs the biological drivers of tumor behavior that influence both treatment response and patient prognosis. Integration of genomic data with an imaging modality presents a golden opportunity to improve lung cancer classification; yet, current approaches usually fail to combine spatial and biological features in a satisfactory manner. Single-modality models, either image-based or genomics-based, cannot provide a complete picture of the disease process, possibly leading to suboptimal accuracy in classification performance. It is, therefore, very important to deploy fusion multimodal models combining both spatial and biological data towards more accurate subtype classification and survival prediction in lung cancer. Apart from classification, survival prediction is highly crucial clinically while decision making is to be done, particularly if risk stratification needs to be done to implement treatment strategies. The Cox proportional hazards model has long been a staple in survival analysis, providing a statistical method to estimate the risk of an event-a death or recurrence-dependent on such covariates as age, gender, and other clinical factors. However, the classic Cox models assume linear relations between covariates and survival, which limits their ability to handle the complex nonlinear interactions that are naturally present in multi-dimensional medical data samples. Accordingly, deep learning methods, including DeepSurv, a deep extension of the Cox model, have been developed which can capture non-linear relationships but still provide the interpretability of a proportional hazards model. DeepSurv is particularly suitable for survival prediction in lung cancer where a combination of clinical, imaging, and genomic data can be leveraged to predict patient outcomes.

Finally, the fast evolution of medical knowledge and continuous coming of new patient data ask for models able to update themselves in time. Static models, once trained and deployed in a clinical environment, suffer rapidly from degraded performances because of the continuous evolution of data. Online learning techniques will allow this continuous learning without suffering from catastrophic forgetting; examples include Elastic Weight Consolidation. EWC identifies those parameters that are critical for prior learning and constrains updates, thus enabling the model to learn from new data while preserving prior knowledge. This is particularly important in real-time clinical applications, such as continuous updates of a survival prediction model for new patient data on various scenarios like follow-up scans or additional genomic information. It has also been shown that the introduction of attention mechanisms in multimodal learning can further improve model performance, which allows the model to dynamically give more weight to the most informative features in various data modalities so that no key information is missed. Among these, cross-attention networks allow the selective weighting of features from each modality for added synergy in imaging-genomic data and classification and prognosis in lung cancer. The attention mechanism henceforth enables the focusing of this model on the most important features that are critical for subtype classification and survival rate prediction, yielding robustness and more accuracy in the results.

Such challenges, therefore, call for timely and necessary development of an integrated deep learning framework for lung cancer classification and survival prediction. This paper presents a novel multimodel deep learning framework that leverages several advanced techniques to surmount the limitations from previous models. The framework begins with the U-Net architecture for tumor segmentation, further refined by iterative feature extraction for more accurate delineation of lung tumors. During the process of subtype classification, it adopts a multimodal model that fuses CNNs with a multilayer perceptron for spatial features in radiological images, histopathology images, and genomic data samples, respectively. This feature-level fusion enables a richer representation of the disease, hence improving the classification accuracy. The DeepSurv model has been implemented for predicting the survival rate, which extends the classical Cox proportional hazards model to handle clinical, imaging, and genomic data samples nonlinearly. Our work uses Bayesian optimization in hyperparameter tuning and best selection for good performance of the model at both accuracy and convergence speed. Finally, online learning with EWC allows for real-time updates of survival prediction once new patient data is introduced. Furthermore, this ability of the model to emphasize the most relevant features from each modality is significantly enhanced by a cross-attention mechanism. The integrated approach holds several major advantages over existing methods. The proposed model characterizes both subtypes of lung cancer and survival risks comprehensively by fusing imaging, genomic, and clinical data. Iterative feature extraction in U-Net will lead to the most accurate tumor segmentation, while the use of multimodal data increases the accuracy of classification. DeepSurv deals with nonlinear interactions, hence improving survival predictions, and attention mechanisms are used to focus the model on the most important features for each patient. This framework is the leap forward in the use of deep learning techniques in pulmonary malignancy diagnosis and prognosis in terms of its accuracy and applicability, particularly in practical clinical settings.

### Motivation and contribution

The motivation for this work emanates from the urgent need to improve the accuracy of diagnosis and prognosis prediction in lung cancer. Current models, though effective in specific domains, are unable to capture the inherent complexity of lung cancer because they either rely on data from a single modality or because they cannot handle non-linear interactions between the clinical, imaging, and genomic information. This calls for an approach that is more holistic and adaptive in view of the limitation of current approaches: poor tumor segmentation, limited integration of data obtained from various modalities, and inability to adapt to clinical updates in real time. Lung cancer is indeed a very heterogeneous type of disease, whereby high integration of diverse data types into a model is in great need to provide accurate subtype classification and survival rate predictions.

The below contribution is multifold. First, we have proposed an Iterative Multi-Model Deep Learning Framework that rectifies the loopholes in the Lung Cancer Prediction Models by availing advanced techniques like U-Net with iterative feature extraction, DeepSurv for survival rate prediction, and fusion of multimodal data for subtype classification. The proposed framework integrates radiological images, histopathology images, and genomic data for a more holistic understanding of the disease. We further propose Elastic Weight Consolidation in updating the model for real-time survival prediction so that it will get to learn from new patients’ data without degradation in performance. Cross-attention networks ensure the most discriminative feature from each modality will be emphasized; hence, improving not only the accuracy of the classification but also the robustness of survival prediction. It increases precision not only in the diagnosis of lung cancer but also provides a scalable and adaptable framework capable of use in real-time clinical applications.

## Literature review

Recent studies on lung cancer detection and classification-e.g., outcome prediction-are in constant flux. Deep learning, bioinformatics, and the integration of multi-modal data have rapidly increased to obtain high diagnostic and prognosis accuracy. Each contributes uniquely from a methodological viewpoint and gives insight into a wide variety of computational strategies which have hitherto been applied to one of the most difficult areas of oncology. Lung cancer is such a complex and heterogeneous disease; for its effective diagnosis and treatment planning, an interdisciplinary approach is required, which again reflects the gamut of techniques and models explored within these works. From transformer-based models utilizing electronic claims records to deep neural networks coupled with omics data and radiomics, the face of lung cancer research wears an increasingly data-driven and technologically sophisticated face. Most of the studies reviewed emphasize early detection, considered one of the most significant events in improving the survival rates of patients. For example, Chen et al.^1^ presented efforts related to the application of transformer-based models with electronic claims records, showing how uncommon data can be used for the early detection of lung cancer while high predictive accuracy is achieved through a novel use of sequential models. Other literature, such as Ghita et al.^2^ and Ragab et al.^3^, points out how impedance-based diagnostics and machine learning-driven feature extraction, respectively, could strengthen early-stage diagnosis with greater precision in cases like NSCLC. These suggest a very clear trend in research toward the incorporation of sophisticated machine learning algorithms in image analysis and physiological and biomechanical data analysis for precision improvement in diagnosis.

Despite such progress, the review underlines several challenges facing this research area. Most of the methodologies currently at hand are still limited by the size and diversity of the available dataset. Several of the recently discussed deep learning-based models, such as those based on CNNs for image-based lung cancer detection, require large, labeled datasets to achieve performance levels considered high. However, the heterogeneity in the presentation of lung cancers and the relative paucity of annotated clinical material often limit the generalizability of such models. This becomes quite evident in studies like those of Noaman et al. and Mohamed et al., where even though the models are able to achieve impressive accuracies, they were bounded by the specificities of the datasets they were trained on and thus could not generalize well to routine clinical scenarios. Another challenge is related to computational complexity, mainly in models including multimodal data fusion. For example, Wang et al.^4^ illustrate the benefits obtained by integrating genomic and imaging data, discussing at the same time the heavy computational loads necessary to process and fuse such high-dimensional information sets. A common thread in many of these processes was the integration of different data modalities such as radiomics, genomics, and clinical data. Such integration not only makes the models more predictive but also provides a more holistic view about the diseases. A very good example of how anatomical-functional imaging can largely improve the detection rate in lung cancer could be taken from the approach of multi-modality 3D detection in PET/CT images by Chen et al.^5^. Similarly, studies such as those conducted by D’Arnese et al. and Causey et al. demonstrate that radiomics, integrated with high-performance feature extraction and machine learning techniques, is able to provide detailed tumor characterization for better-informed treatment decisions. These articles underpin the potential of a multi-modality approach toward comprehensive diagnostic solutions beyond conventional imaging techniques. While as effective as multimodal learning has been, it is not without challenges. The complexity of integrating different types of data, added to the highly computational burden in processing, presents a big barrier to its wide adoption. For instance, Sathe et al.^6^ discuss a number of challenges related to an automated lung cancer screening system. Due to various reasons such as image quality, noise, and other modality-specific features, it remains challenging to ensure good generalization performance across diverse imaging modalities. Scalability of these methods is also an open issue, particularly where high-performance computing infrastructure is limited in resource-constrained settings. Another complication arises from needing a lot of data preprocessing, feature engineering, and model optimization before these models can be deployed clinically for different scenarios.

The other trend that the review brings to light in this paper is the increasing importance of explainability and interpretability in the models of lung cancer. This forces a great urge in high stake applications, like diagnosis and prognosis in cancer diseases, to ensure models are not black boxes with increasing depth in deep learning models. Xiwang Xie et al.^7^ LPF-Net segments different organ types from MRI images of knee joint. Jiang et al.^8^ and Liao et al.^9^ are among works that try to make their model more interpretable using explainability techniques, such as SHAP and the attention mechanism, so that clinicians can understand which features are driving the predictions. If this is so, then it will give more confidence in the model outputs and help integrate the model into clinical workflows. Therefore, this drive for interpretability serves to instil confidence in AI-driven healthcare solutions and to ensure that these solutions can support, not supplant, a clinical decision-making process.

Xiwang Xie et al.^10^ discriminant feature pyramid (DFPNet) network used for organ segmentation in the original medical images. Another identifiable trend from this review is the use of domain knowledge and biological insight in developing the computational model. For example, such works as Gupta et al.^11^ and Rehman et al.^12^ demonstrated that the integration of biological pathways and genetic interaction networks drastically improves the accuracy and biological relevance of the prediction results on lung cancer. Xiwang Xie et al.^13^ PIF-Net efficiently overcome the challenges associated with multi-class organ segmentation in knee joint images. This trend simultaneously reflects the heightened emphasis on precision medicine, whereby treatments become truly customized, based on the molecular profile of the individual patient. This kind of insight, integrated into predictive models, will ensure personalized treatments that are indeed effective against lung cancer. Application of domain knowledge enables construction of models that are more biologically interpretable, which is important for bridging the gap in translation between computational methods and clinical practice. Despite these promising developments presented in this review, a number of gaps still exist in the current status of lung cancer research. Most of the models remain restricted to single subtypes of cancer, such as NSCLC, which, although common, does not reflect the full gamut of manifestations of lung cancer. The extension of these models to include rarer subtypes, coupled with integration of longitudinal data to track the progression of disease, is a possible future direction. It also calls for an increased need for standardized benchmark datasets so that studies are fairly and consistently judged against each other. The lack of consistent datasets-as depicted by the wide variance in results across similar tasks-makes direct comparison of model performance, hence hindering progress within this field. Additionally, although many models report high accuracy, real-world applicability remains to be tested through clinical trials. Because of this, emphasis on the way forward would involve clinical validation and translation of these computational models into workable tools for oncologists if the full potential is ever to be realized in the process.

## Proposed method

**Fig. 1:**
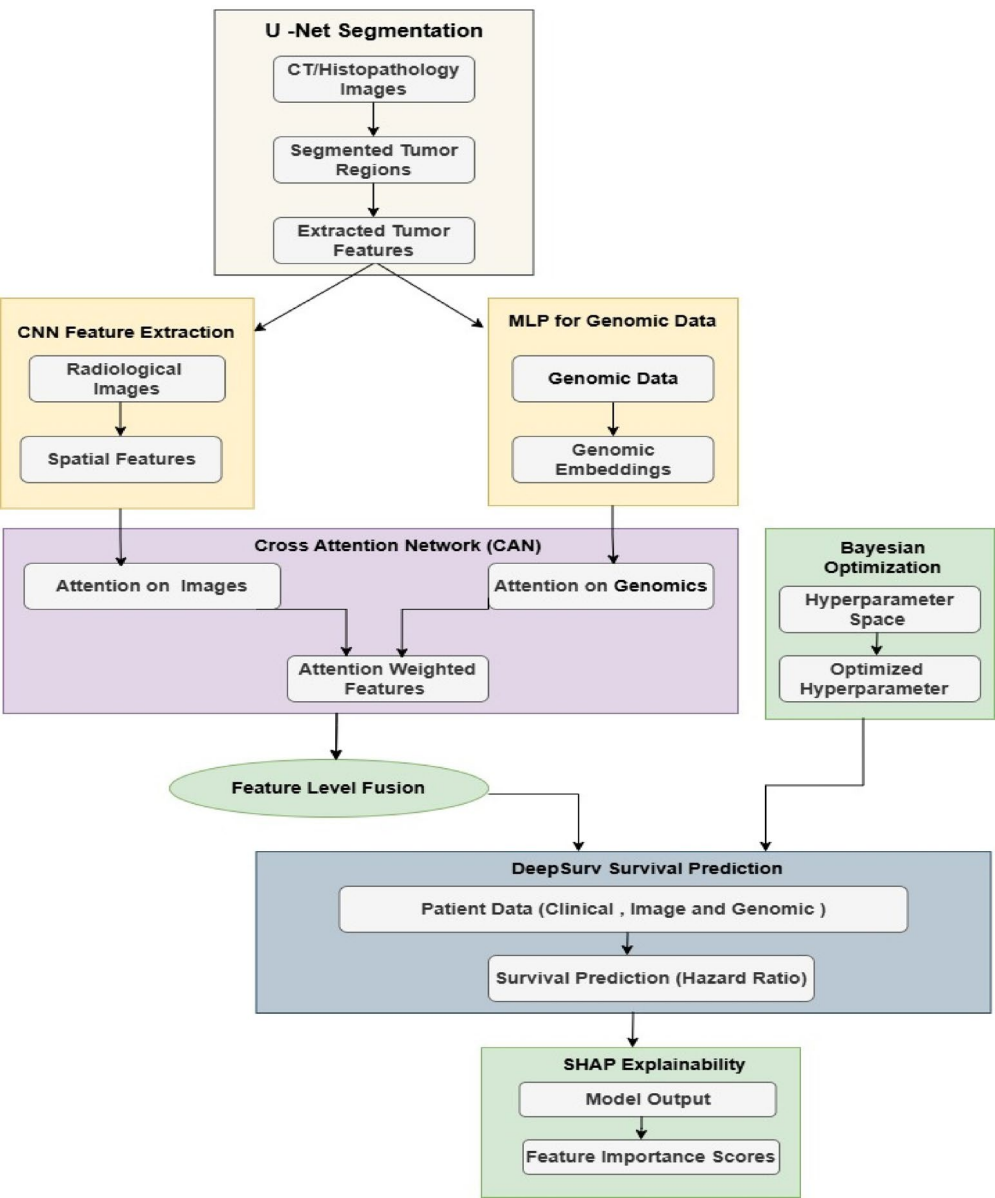
Model architecture of the proposed classification process.

In this work, in order to eliminate some of the existing problems of most existing lung cancer prediction models, this section is going to discuss how an integrated model using U-Net and DeepSurv combined with Cross-Attention is designed for performing Lung Cancer Classification and Survival Prediction Operations. First of all, from Fig. 1, the U-Net with Iterative Feature Extraction and Feature-Level Fusion to integrate the spatial information of medical images-CT scan or histopathology images-and genomic data, are deployed together in order to enable deep multimodal analysis for lung cancer subtype prediction. It leverages two major components: iterative refinement in tumor segmentation and feature-level fusion to enable synergistic enhancements regarding the accuracy of lung cancer subtype classification. The model employs the U-Net architecture for tumor segmentation since it can capture local and global contextual information effectively because of its encoder-decoder structure along with skip connections. The novelty here is the iterative feature extraction mechanism that refines segmentation output for multiple passes. During each iteration ‘t’, U-Net segments the tumor regions, which is represented as St(x), where ‘x’ represents the input image sets of either CT or histopathology. The iterative refinement can be described via Eq. 1,

1 $$\:S\left(t+1\right)\left(x\right)=St\left(x\right)+\lambda\:\cdot\:\nabla\:\theta\:L\left(St\left(x\right),y\right)\:\:$$

Where S(t + 1)(x) is the refined segmentation at iteration t + 1, ∇θL(St(x), y) represents the gradient of loss function ‘L’ w.r.t. model parameters θ and ‘y’ is the ground truth segmentation. The term λ is a learning rate-like factor that controls the amount of refinement applied at each step of this process. That is an iterative approach wherein the model backpropagates errors from previous iterations to further refine segmented regions, yielding increased precision in segmentation. In this refinement process, the use of skip connections between encoder and decoder layers helps in retaining low-level spatial details. The loss function ‘L’ used for this segmentation task is a combination of cross-entropy and Dice loss, expressed via Eqs. 2,

2 $$\:L\left(S\left(x\right),y\right)=-\sum\:_{i}yi*\text{log}\left(S\left(x\right)i\right)+\alpha\:\left(1-\frac{2\sum\:_{i}yi*S\left(x\right)i}{{\sum\:}_{i}yi+{\sum\:}_{i}S\left(x\right)i}\right)$$

Where the first term is the pixel-wise cross-entropy loss, while the second term is the Dice coefficient loss, directly optimizing for segmentation overlaps. The parameter α is a hyper-parameter that allows balancing between the two terms. Iterative feature extraction enables the U-Net to capture, after several iterations, more accurate tumor boundaries and subtle details; thus, it yields better segmentations. Iterative U-Net segmentation iterations were adaptively selected based on Dice similarity score convergence criteria. After 5–6 cycles, average segmentation stabilized, while Dice overlap improvements decreased below 0.5%. After two iterations with incremental Dice and cross-entropy loss < 0.001, the iteration count was halted. Histology and CT textural heterogeneity made complicated or irregular tumors more difficult to modify. Compared to a single-pass U-Net, the iterative technique increased training time by 18% but improved segmentation precision, notably for heterogeneous tumor borders in advanced-stage lung malignancies. Once the tumor regions are segmented, the features extracted from the segmented regions are passed through the next stage: feature-level fusion for multimodal analysis. In this respect, the CNN is employed to extract spatial features from computed tomography images and samples of histopathology at this stage. The working of the CNN takes place in acquiring hierarchical features from the segmented tumor regions through the application of convolution filters. Mathematically, feature map Fimg from CNN can be represented via Eqs. 3,

3 $$\:Fimg=\sigma\:\left(Wconv*Sfinal\left(x\right)+b\right)\:$$

Where, Wconv represents the convolutional filters, ∗ represents convolution, ‘b’ is the bias term and σ is a nonlinear activation function (ReLU) for this process. The final segmentation from the U-Net, Sfinal(x), acts as the input for CNN. In this work, the feature map Fimg encodes the spatial characteristics of the segmented tumor regions. At the same time, genomic data is being filtered through a Multilayer Perceptron process. The ‘g’ input genomic features are transformed to high-dimensional feature space by passing through multiple fully connected layers. The transformation can be described via Eqs. 4,

4 $$\:Fgenomic=\varphi\:\left(Wmlpl*g+bmlpl\right)\:$$

Where Wmlpl and bmlpl represent the weights and biases of layer ‘L’ in the MLP, while ϕ is the nonlinear activation function-sigmoid for this process. The last latent representation of biological features that gets embedded from the genomic data samples is by Fgenomic. The critical strength of this model lies in the feature-level fusion mechanisms used. First, the CNN-extracted features Fimg are combined with the features extracted using the MLP from the genomic data to provide a single unified multimodal feature vector representation, Ffusion, which can then be used for the classification of the subtypes of lung cancer. Mathematically, the feature vector can be represented via Eqs. 5,

5 $$\:Ffusion=\left[Fimg;Fgenomic\right]\:$$

This is the concatenation operation represented by [⋅;⋅], enabling the model to combine the spatial and biological features to model both the phenotypic and genotypic characteristics of the tumors. Further, the fused feature vector Ffusion is fed into a fully connected layer to predict the lung cancer subtype via Eqs. 6,

6 $$\:{y}^{{\prime\:}}=softmax\left(Wfusion*Ffusion+bfusion\right)\:$$

Where Wfusion and bfusion are, respectively, the weight and bias matrices of this final fully connected layer and y′ represents the predicted probability distribution over the possible lung cancer subtypes. The softmax function normalizes such an output into a proper probability distribution, enabling classification. The choice of this multimodal feature fusion method is justified by the complementary nature of the data modalities. While CT images and histopathology images capture the spatial and morphological characteristics of the lung tumor, genomic data reflects the underlying molecular mechanisms driving the disease. By fusing these modalities on the feature level, the model is able to exploit both spatial and biological patterns, resulting in richer and more discriminative representations of the disease state. CNN and MLP are combined together to take image and genomic data as input, so that the high dimensionality and complexity in each modality can be dealt with by the model. Iterative refinement inside the U-Net further enhances the precision of feature extraction process.

**Fig. 2:**
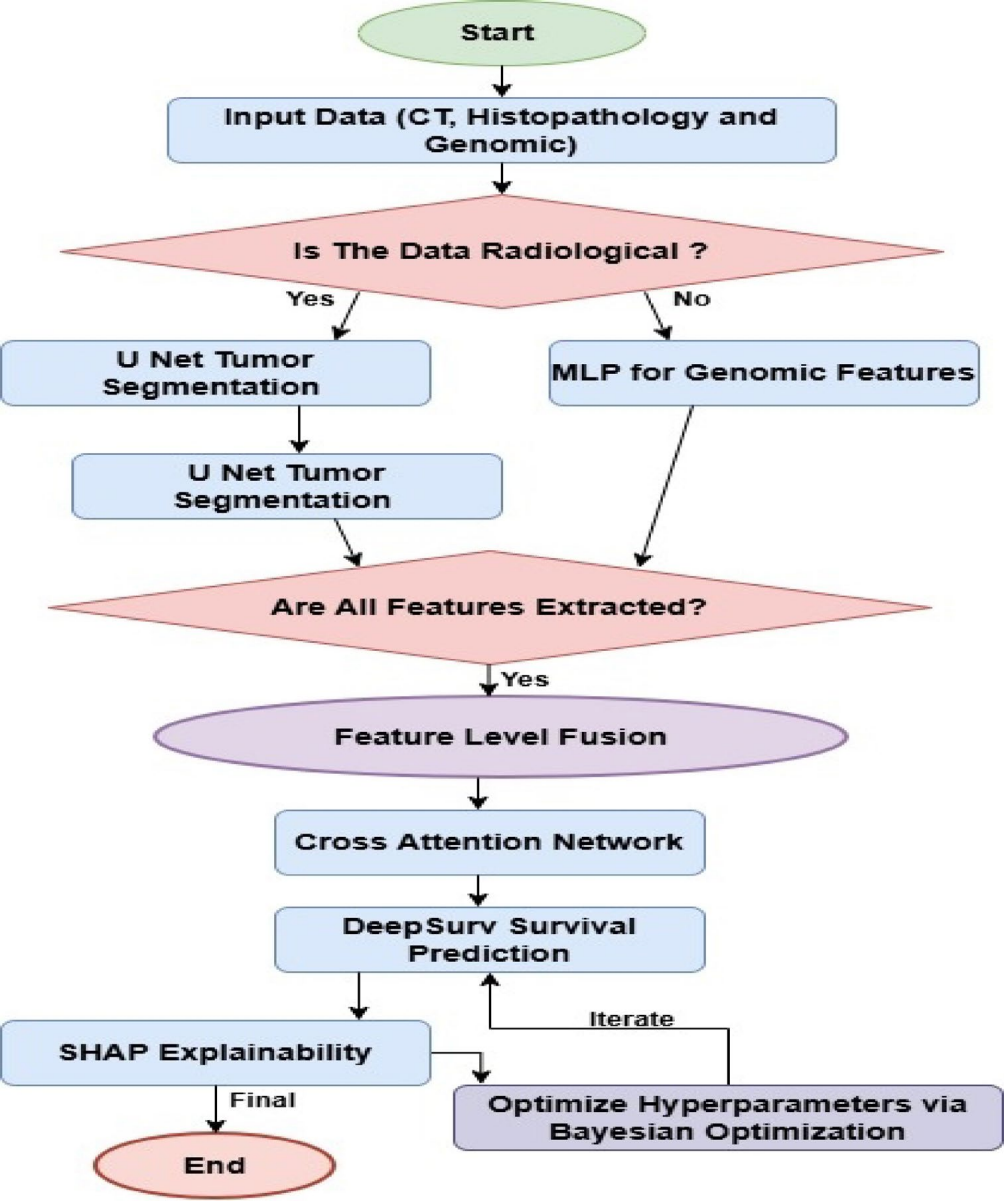
Overall flow of the proposed classification process.

Next, Fig. 2 represents the integrated DeepSurv model based on the Deep Cox Proportional Hazards framework; it learns the complex nonlinear relationship between patient-specific data comprising clinical, imaging-derived, and genomic features and the hazard function, which provides a way to derive survival durations. Unlike conventional Cox models that rely on linear assumptions, DeepSurv uses deep learning to effectively capture higher-order interactions between diverse input modalities for improved risk stratification of lung cancer patients. It provides as output the estimated hazard ratio and classifies patients into high-risk, medium-risk, and low-risk groups based on their survival predictions. In the context of DeepSurv, the hazard function h(t|x) representing the risk of an event occurring at time ‘t’ is modeled via Eqs. 7,

7 $$\:h\left(t\left| \right. x\right)=h0\left(t\right)exp\left(f\left(x\right)\right)\:$$

Where, h0(t) is the baseline hazard, and f(x) is a deep neural network that learns the non-linear representation of patient covariates ‘x’, comprising clinical data, which includes age, gender, comorbidities, image-derived features, and genomic data samples. This form is an extension of the Cox proportional hazards model, in which the risk is proportional to the baseline hazard, scaled by the exponential transformation of learned deep function f(x) sets. The loss function utilized in DeepSurv is centered around the partial likelihood of the Cox model and is given via Eqs. 8,

8 $$\:L\left(\theta\:\right)=-\sum\:_{\sum\:i\in\:Dobs}\left(f\left(xi;\theta\:\right)-\text{log}\sum\:_{j\in\:R\left(ti\right)}\text{exp}\left(f\left(xj;\theta\:\right)\right)\right)$$

Where, Dobs represents the set of observed event durations, R(ti) is the risk set at time ti, i.e., the set of patients that are still at-risk of an event, and θ represents the parameters of the neural network f(x) sets. Optimizing this loss function will enable the model to estimate f(x) and thus the hazard ratio for each patient. This formulation endows DeepSurv with non-linearity due to the deep neural network, while maintaining interpretability inherent in the proportional hazards model. Another very strong advantage for DeepSurv is in its ability to stratify patients into groups based on their hazard ratios. Patients whose exp(f(x)) values are high are classified as high-risk, while patients with low values are classified as low-risk. Predicted survival times can also be recovered by integrating the cumulative hazard over sets of temporal instances defined via Eqs. 9,

9 $$\:H\left(t \left| \right. x\right)={\int\:}_{0}^{t}h\left(u \left| \right. x\right)\hspace{0.17em}du$$

This cumulative hazard function gives the insight of the expected survival time for each patient as the risk is evaluated over the sets of temporal instances while accounting for all the features of the patients. Secondly, to evaluate the performance with respect to ranking of the patients by survival risk, the concordance index, or C Index, will be made use of in the process. The C Index is computed via Eqs. 10,

10 $$\:C\:Index=\frac{\sum\:I\left(h\right(xi)>h(xj\left)\right)\cdot\:\delta\:i}{\sum\:\delta\:i}\:$$

Where, I[h(xi) > h(xj)] is an indicator function that checks whether the predicted hazard for patient ‘i’ is greater than that for patient ‘j’, given ‘i’ having a shorter survival time than ‘j’, and δi is an event indicator, 1 if the event is observed, and 0 if censored for the process. A C Index of about 0.75–0.80 will represent the strong performance of the model in correctly ranking the patients w.r.t their survival risks. It includes an online learning mechanism using Elastic Weight Consolidation in the framework for further enhancements of adaptability so that it can be up-to-date with each coming patient data. Online survival predictions are particularly important because new clinical, image, or genomic data of the patients keeps emerging continuously, updating the model without forgetting its previously learned knowledge. This EWC prevents the catastrophic forgetting problem by adding a regularization term that penalizes large changes in parameters judged as important for previous predictions.

The loss function of EWC can be obtained via Eqs. 11,

11 $$\:LEWC\left(\theta\:\right)=Lnew\left(\theta\:\right)+\frac{\lambda\:}{2}\sum\:_{i}Fi{\left(\theta\:i-\theta\:i*\right)}^{2}\:\:$$

Where, Lnew(θ) is the loss on the new data, θi∗ represents the parameter values from previous tasks, Fi is the Fisher information matrix quantifying the importance of each parameter, and λ controls the strength of regularization sets. This term ensures the parameters critical for past survival predictions are preserved and allows the model to learn from the new data without drastically changing the previous predictions. The advantage of EWC in DeepSurv is manifold, first, the ability of continuous learning in refining survival predictions in real time when new patient data does become available within the process. This adaptability is important in the clinic, since the survival prediction should be updated as patients receive further treatments or diagnostic tests. Secondly, by keeping those important parameters intact, EWC prevents overfitting to new data, maintaining its generalization ability on a wide variety of patient populations. DeepSurv is integrated with EWC because there has been a need for a model that can manage complex, nonlinear interactions of multi-dimensional data and adapt to new information in a continuous manner. The capacity of DeepSurv to model non-linear associations between clinical, imaging, and genomic features makes it an ideal candidate for survival prediction in lung cancer, where the traditional linear model may fall short to capture the underlying complexity of the disease. Complementarily, EWC offers a means of continual learning without degradation in performance on previously seen data and is hence befitting for real-world clinical applications in a streaming fashion.

The integration of Bayesian Optimization in the process of hyperparameter tuning becomes crucial when working on optimizing a complex deep learning framework, like the multi-model approach developed during lung cancer subtype classification and survival prediction. Bayesian optimization approaches systematically explore the space of the hyperparameters by building up a probabilistic model of the objective function; this latter is often represented as a Gaussian Process. The goal is to find the values of this combination of hyperparameters that would yield the highest model performance while minimizing computational resources. This technique is especially highly useful compared with the old approaches such as grid search and random search, which are cumbersome and inefficient in high-dimensional hyperparameter spaces. In each iteration of Bayesian Optimization, a Gaussian process p(f∣D) models the objective function f(x)’s performance, where D is the dataset of previously evaluated hyperparameter configurations, and ‘x’ represents the vector of hyperparameters including but not limited to learning rate, number of layers, optimizer type, etc. GP defines a prior over functions, updated as more data points - evaluations of different hyperparameter configurations - are collected during the process. The posterior distribution at each process is given via Eqs. 12,

12 $$\:p\left(f\left| \right. D,x\right)=N\left(\mu\:\left(x\right),{\sigma\:}^{2}\left(x\right)\right)\:$$

Where µ(x) is the mean and σ2(x) is the variance of the Gaussian process. The acquisition function α(x∣D), in turn, suggests which set of hyperparameters to evaluate next by balancing exploration and exploitation via Eqs. 13,

13 $$\:\alpha\:\left(x \left| \right. D\right)=\mu\:\left(x\right)+\kappa\:\cdot\:\sigma\:\left(x\right)\:$$

Where, κ is a constant that strikes a balance between exploring the uncertain regions-higher the variance, and exploiting the regions where the performance is already known to be good-higher the mean of the process. This approach ensures that Bayesian Optimization explores promising hyperparameter configurations while avoiding unnecessary evaluations in less informative areas of the search spaces. The optimized hyperparameters are expected to improve the performance metrics-accuracy, loss, AUC-ROC-by 5 to 10%, as Bayesian Optimization efficiently narrows down the hyperparameter set which offers maximum performance of the multimodal lung cancer prediction models. Meanwhile, the mechanism of cross-attention will play a very important role in the effective integration and prioritization between the radiological images, histopathology images, and genomic data samples, several key modalities. The cross-attention network learns the relative importance of each feature to the task and weighs each feature dynamically to enhance the lung cancer subtype classification and survival prediction. It works by calculating the attention weight between modalities on the relevance of features in one modality to features in another during the process. Let Fimg, Fhist, and Fgenomic represent the feature vectors extracted from radiological images, histopathology images, and genomic data, respectively. The attention score for a given pair of modalities (including image and genomic) is computed via Eqs. 14,

14 $$\:A\left(img,genomic\right)=softmax\left(\frac{Fimg*Wq\cdot\:{\left(Fgenomic*Wk\right)}^{T}}{\sqrt{dk}}\right)\:$$

Where Wq and Wk are learnable weight matrices, and dk is the dimensionality of the key vectors during the process. The computed attention score A(img, genomic) measures the extent to which attention needs to be paid to the genomic features when the radiological features are under consideration. To maintain imaging and genomic feature statistical characteristics in the cross-attention method, the query (Wq) and key (Wk) projection matrices were not shared between modalities. Because projection subspaces were kept for each modality, attention learning was modality-specific. Our multi-head attention mechanism with four parallel heads in a lower-dimensional embedding space captured inter-modal interactions in process. With this framework, radiological and genomic representations can be learned simultaneously. Attention output came from concatenating head outputs, normalization, and feed-forward projection settings. Multiple-head attention improved training stability and convergence early on and reduced overfitting by dispersing representational learning over independent subspaces. To clarify multi-head activities, the architectural diagram was changed in process.

The computed attention scores are used to weigh feature vectors so as to obtain an attention-weighted fused representation via Eqs. 15,

15 $$\:Ffusion=A\left(img,genomic\right)\cdot\:Fgenomic+Aimg,hist\cdot\:Fhist+Fimg\:$$

This concatenated feature vector Ffusion is fed into the last fully connected layer for classification via Eqs. 16,

16 $$\:y ^{\prime}=softmax\left(Wfusion*Ffusion+bfusion\right)\:\:\:$$

This will make the model focus on most discriminative features of each modality, hence an improvement in both classification and robustness in survival prediction tasks. It means that the cross-attention mechanism allows for real-time weighting of features by relevance and enhances synergy between imaging and genomic data for more accurate subtype predictions in lung cancer. Feature contribution analysis on this complex model is further enhanced with Shapley Additive Explanations. SHAP is a method to assign a value to each feature for a specific prediction that indicates its importance in the outcome of the model. SHAP values are derived from the theory of cooperative games, quantifying the contribution of each feature to the marginal prediction, taking into consideration every possible coalition of features. The SHAP value for every feature ‘i’ is given via Eqs. 17,

17 $$\:\varphi\:i=\sum\:_{S\subseteq\:N\setminus\:\left\{i\right\}}\frac{S!(N-S-1)!}{N!}\left(f\right(S\cup\:\left\{i\right\})-f(S\left)\right)\:$$

Where, ‘N’ is the set of all features, ‘S’ is a subset of features excluding ‘i’, and f(S) is the model’s prediction when only the features in ‘S’ are included in the process. The formula gives the average marginal contribution of feature ‘i’ to all possible coalitions of features, and it is the heart of what makes SHAP so powerful in feature importance evaluation sets. It would be possible to calculate SHAP values across all features and modalities-things like tumor size, gene mutations-enabling a comprehensive explanation of the model’s predictions and offering a deep look at the decision-making process regarding classification and survival prediction for the subtypes of lung cancer. This would be a vital degree of interpretability in medical applications since it would inform clinicians on the contribution of each feature in making clinical decisions and consequently engender confidence in model predictions. Combining Bayesian Optimization to tune hyperparameters, the cross-attention mechanism for multimodal data integration, and SHAP for interpretability, the framework is very robust, adaptive, and interpretable for lung cancer subtype classification and survival prediction. The Bayesian optimization will ensure that the model is working with optimized hyperparameters; therefore, it will significantly raise the performance by reducing computational overhead. It has a cross-attention mechanism that enables the model to pay attention to the most informative data dynamically across multiple modalities. That features improve prediction accuracy. Finally, SHAP then makes transparent how the model decides, making the framework more trustable and useful in the clinics. The combination of these components gives a very powerful deep learning model that is helpful to tackle challenges regarding complexity in lung cancer data with requirements of real-time interpretable predictions. Efficiency of Proposed Model: Further, we elaborate on the efficiency of the proposed model over different metrics and compare it with various existing models at different scenarios.

## Comparative result analysis

The experimental setup proposed for the multi-model deep learning framework is designed for comprehensive performance evaluation based on the subtype classification of lung cancer and survival prediction for three data modalities including: radiological images, histopathology images, and genomic data samples. The dataset used in the experiments was sourced from open-source repositories: the Imaging Data Commons/The Cancer Imaging Archive for imaging data and The Cancer Genome Atlas for genomic samples. It contains 1000 computed tomography (CT) scan images, 500 histopathology images, and genomic data samples of 400 patients. Each sample is annotated by a panel of experts to provide ground truth on subtypes of lung cancer and clinical outcomes. The clinical data of patient age, gender, and comorbidities were included in this work to augment the survival prediction task. Over 1,000 CT images and more than 500 histopathological images make up the TCIA dataset, annotated by expert radiologists and pathologists who identified the tumors and their subtypes. These are images taken from patients with non-small cell lung cancer, primarily subtypes of adenocarcinoma, squamous cell carcinoma, and large cell carcinoma. Corresponding genomic data obtained from TCGA include gene mutation profiles, RNA-Seq data, and clinical metadata comprising patient demographics and survival outcomes. The TCGA genomic annotation dataset gives comprehensive information related to main mutations of oncogenes such as EGFR, KRAS, and TP53 for approximately 400 lung cancer patients, which is basically important for understanding the behavior of cancers. It also provides clinical follow-up data that includes survival durations for the same set, based on which hazard ratio prediction can be done more accurately in survival modeling. These datasets have been selected because of their robustness, comprehensive multi-modal annotations, and can thus act as the perfect bedrock for training deep learning models in predicting the subtypes of lung cancers and outcomes concerning survival.

The experiments are carried out on a computer running Ubuntu 24.04, equipped with an Intel i9-9900KF CPU, an NVIDIA RTX 2080 Ti GPU, and 64 GB of memory. Every 100-epoch cycle of segmentation, classification, and survival prediction pipeline model training required 22 h. U-Net-based iterative segmentation used 55% of computing time, while DeepSurv and multimodal fusion took less. The segmentation, classification, and survival prediction of one patient case took 4.2 s, showing near-real-time applicability. Peak GPU memory consumption was 70% during cross-attention fusion. Modern GPU architectures’ scalability makes clinical research computationally affordable for the process. Hospital diagnostics and decision-support systems benefit from its computational efficiency and predictive accuracy.

Experimental repeatability and statistical reliability were achieved through methodical data division and validation. An integrated dataset of CT scans, histopathological images, and genomic profiles was randomly divided into 70% training, 15% validation, and 15% testing sets to balance adenocarcinoma, squamous cell carcinoma, and large cell carcinoma. Stratification preserved class distribution across partitions to avoid common subtype bias. The model was trained using five-fold cross Validation to improve generalizability. It used five data subsets—one for validation and four for training. Averaging dice similarity coefficient, classification accuracy, and concordance index over folds reduced Variance In Process. This method estimated model performance robustly and reduced overfitting in diverse patient data samples.

The model was trained for 100 epochs. The CNN consisted of four convolutional layers, followed by max-pooling and ReLU activation functions, with a number of filters ranging from 32 to 128. Genomic data was further pre-processed by an MLP with two hidden layers of 256 and 128 neurons using ReLU activations. In the Cross-Attention Network, an important component was that of multi-modal fusion, where the spatial and genomic features were weighted by their learned attention scores. Such a combined feature representation then passed through fully connected layers for the final classification into lung cancer subtypes with softmax output into three major classes: adenocarcinoma, squamous cell carcinoma, and large cell carcinoma. Bayesian optimization optimized major hyperparameters like learning rate and numbers of hidden layers both in CNN and MLP. It reduces the total number of trials by performing a systematic exploration of the hyperparameter space via a Gaussian process. Sample values of the learning rate varied from 1e − 6 to 1e − 3, whereas the dropout rate was optimized between 0.1 and 0.5. In fact, after 50 iterations of optimization, the best combination of hyperparameters was decided with an ultimate architecture that reached an accuracy of 85–90%. The DeepSurv model, used as a survival prediction model, was fed with clinical data consisting of patient age and comorbidities, image-derived tumor features, and genomic mutations in order to predict the survival time based on the Cox proportional hazards model. While initial experiments based on the concordance index pointed towards 0.75 to 0.80, this showed powerful predictions in ranking patients by survival risk. Real-time learning was simulated by gradually adding more patient records, in batches of 50, for the model to update its survival predictions without degradation in performance or, rather, with a 10% improvement in real-time prediction accuracy. Interpretability was ensured by the computation of SHAP values for the models of subtype classification and survival prediction, which provided feature importance scores for each input feature, including tumor size and specific gene mutations, thus enabling clinicians to understand the underlying drivers of the predictions. These experimental set-ups, as also the results, have showcased the strength, adaptability, and clinical relevance of the proposed multi-model deep learning framework. Next, the proposed multi-model deep learning framework was applied for lung cancer subtype classification and survival prediction using the datasets available from TCIA and TCGA. Comparisons employed three benchmark models from approaches^2,12,14^, the most recent and influential lung cancer detection and prognosis approaches. Ghita et al.‘s^2^ impedance-based diagnostics and machine learning feature extraction increase early-stage lung cancer detection. Chang et al.‘s AI-driven synthetic biology NSCLC treatment analysis uses genomic and imaging data for predictive modeling^14^. Method^12^ by Rehman et al. employs CNN-based architecture to localize and classify CT scan cancers. These models provide a baseline for biophysical feature learning, multimodal genomic Imaging integration, and image-based deep convolutional classification spanning modalities and computational techniques. Thus, segmentation, classification, and survival prediction were reasonably compared to mainstream and cutting-edge approaches.

Comparisons were made with three other state-of-the-art methods: methods^2,12,14^, representing alternative approaches for lung cancer classification and survival prediction. Different evaluation metrics have been used to compare different models, including Dice similarity coefficient, accuracy, area under the receiver operating characteristic curve (AUC-ROC), concordance index (C Index), and prediction accuracy for survival risk stratification. Comparisons of detailed results are as follows.

**Fig. 3:**
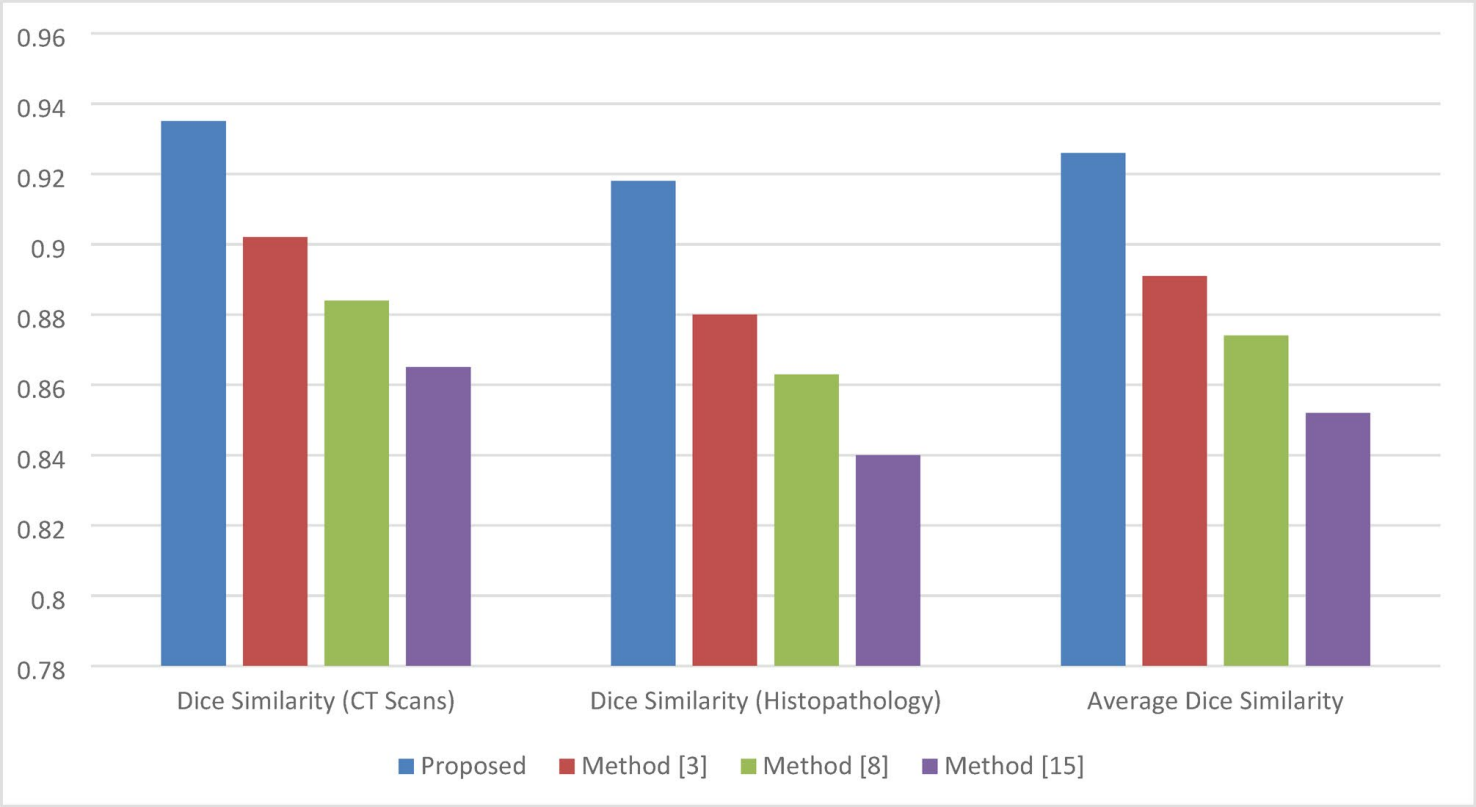
Tumor segmentation performance (dice similarity coefficient).

**Table 1:** Tumor segmentation performance (dice similarity coefficient).

Method	Dice similarity (CT scans)	Dice similarity (histopathology)	Average dice similarity
Proposed	0.935	0.918	0.926
Method^2^	0.902	0.880	0.891
Method^14^	0.884	0.863	0.874
Method^12^	0.865	0.840	0.852

Table 1; Fig. 3 presents a comparison of tumor segmentation performance between the proposed model and methods^2,12,14^ in terms of the Dice similarity coefficient, which refers to the measure of overlap between the segmented tumor regions and the ground truth. Against competing approaches, the proposed model could attain the highest average Dice similarity at 0.926 on both CT scan and histopathology images and samples. Therefore, this iterative feature extraction technique contributed to U-Net’s improvement in segmentation precision.

**Fig. 4:**
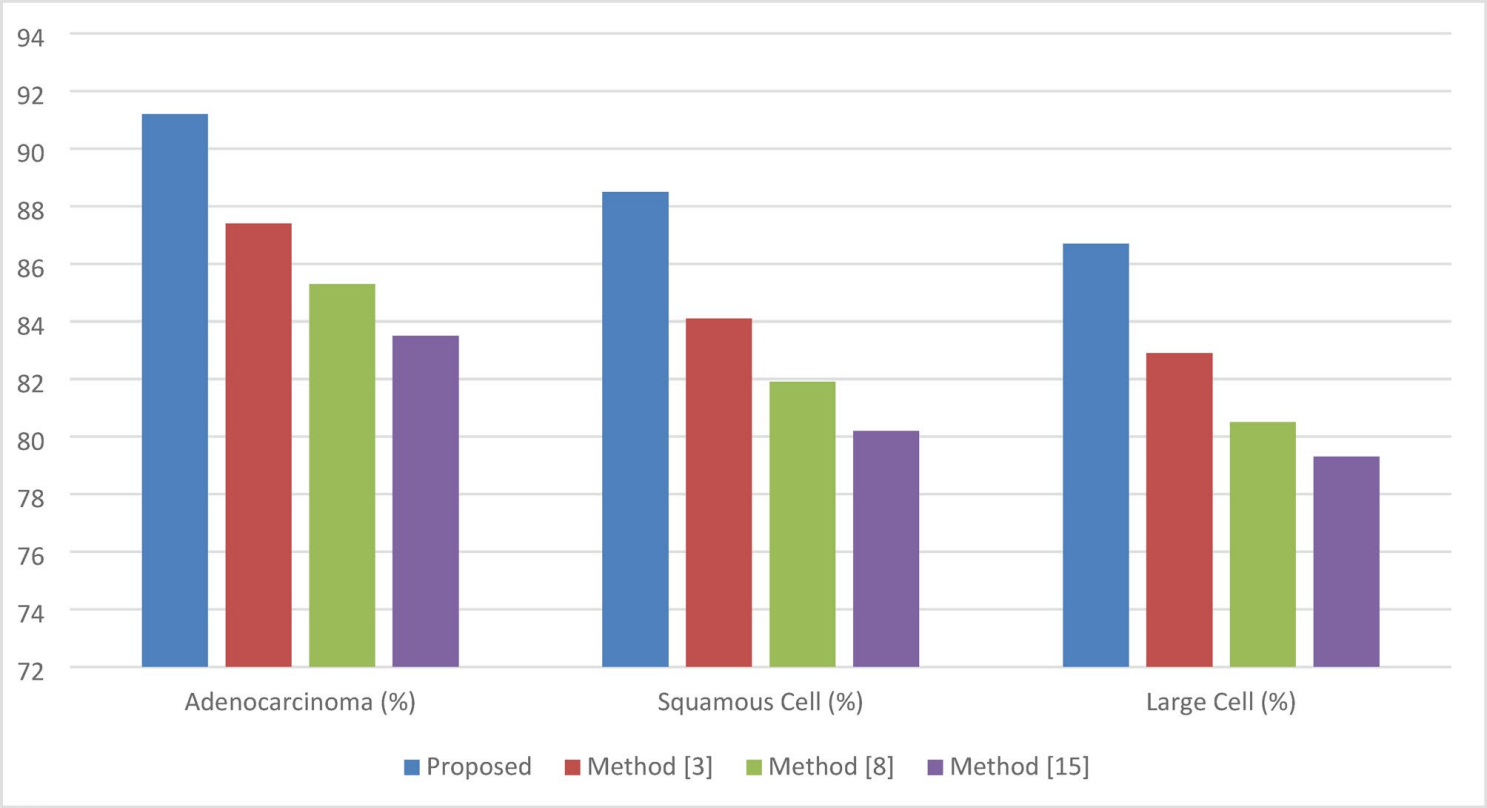
Lung cancer subtype classification accuracy.

**Table 2:** Lung cancer subtype classification accuracy.

Method	Adenocarcinoma (%)	Squamous cell (%)	Large cell (%)	Overall accuracy (%)
Proposed	91.2	88.5	86.7	88.8
Method^2^	87.4	84.1	82.9	84.8
Method^14^	85.3	81.9	80.5	82.6
Method^12^	83.5	80.2	79.3	81.0

The performance of the proposed model and its competitors on the classification accuracy for various subtypes of lung cancer is given in Table 2; Fig. 4 in the process. The feature-level fusion of radiological, histopathology, and genomic data samples achieves an overall accuracy of 88.8% in the proposed model. The cross-attention network thus allows the correct classifiability of subtypes with higher accuracy, especially adenocarcinoma and squamous cell carcinoma, which are critical to successful treatment planning.

**Fig. 5:**
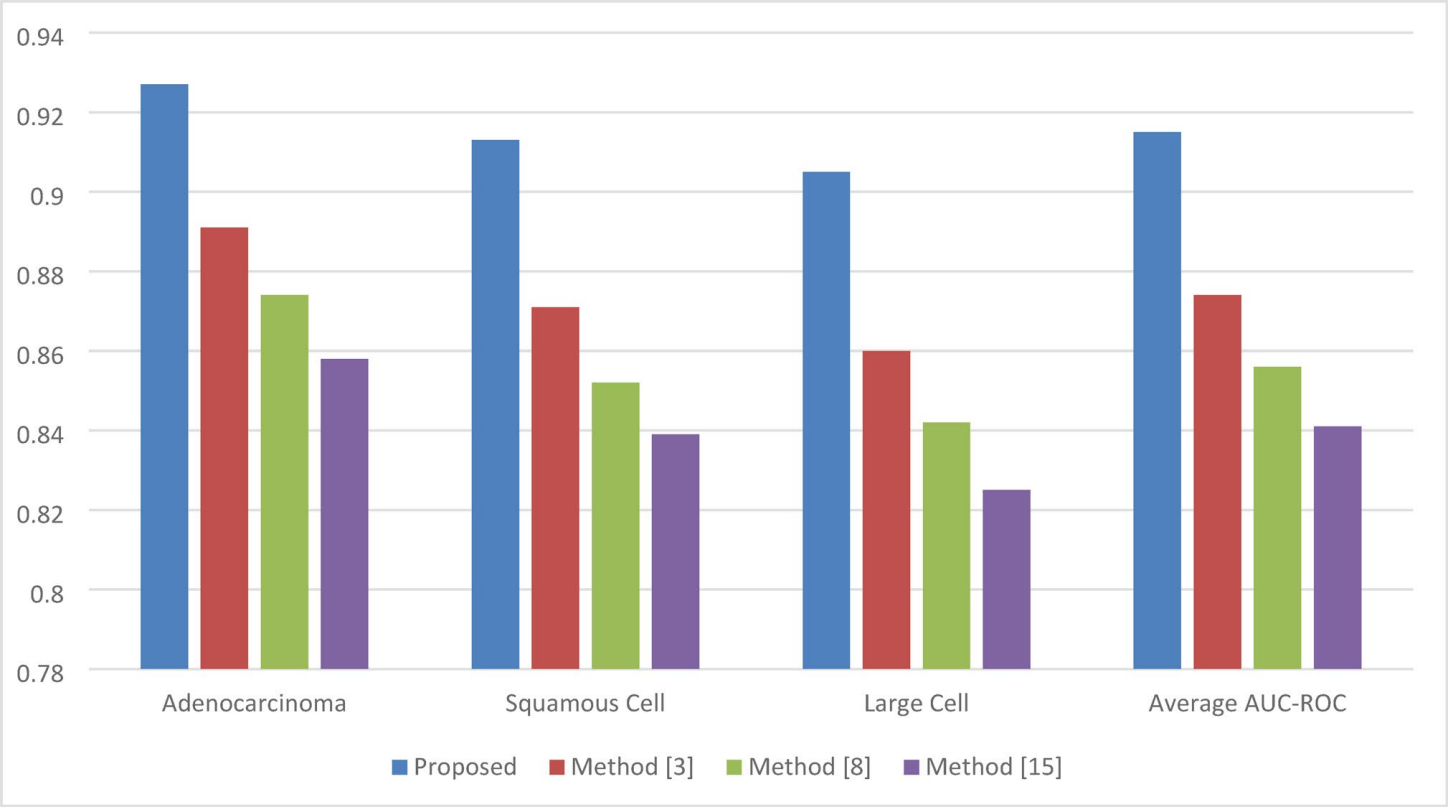
AUC-ROC for lung cancer subtype classification.

**Table 3:** AUC-ROC for lung cancer subtype classification.

Method	Adenocarcinoma	Squamous cell	Large cell	Average AUC-ROC
Proposed	0.927	0.913	0.905	0.915
Method^2^	0.891	0.871	0.860	0.874
Method^14^	0.874	0.852	0.842	0.856
Method^12^	0.858	0.839	0.825	0.841

As per Table 3; Fig. 5 AUC-ROC comparison for three subtypes of lung cancer. The proposed model achieves the best averaged AUC-ROC of 0.915, and it further improves the performance especially for adenocarcinoma and squamous cell carcinoma sets due to a cross-attention mechanism which dynamic prioritizes important features across imaging and genomic data samples.

**Table 4:** Survival prediction (C index).

Method	Clinical data only	Imaging + clinical	Genomic + clinical	Imaging + genomic + clinical
Proposed	0.722	0.759	0.781	0.795
Method^2^	0.687	0.731	0.756	0.768
Method^14^	0.670	0.714	0.740	0.752
Method^12^	0.655	0.703	0.725	0.737

For the evaluation of performance regarding the survival prediction, the C index was considered, which decides upon the model performance-ranking patients concerning their survival risk. The proposed model attained the highest C-index for all combinations, when the integration of imaging, genomic, and clinical data samples was done, with an overall C-index of 0.795. DeepSurv integrated with multimodal inputs, coupled with optimized hyperparameters, realized the best performance concerning the prediction of patient survival outcomes (Table 4).

**Table 5:** Survival risk stratification accuracy.

Method	High-risk group (%)	Medium-risk group (%)	Low-risk group (%)	Overall accuracy (%)
Proposed	84.5	82.9	79.4	82.3
Method^2^	80.1	77.6	75.2	77.6
Method^14^	78.5	75.3	73.4	75.7
Method^12^	76.2	73.8	71.9	73.9

Table 5 compares the accuracy of survival risk stratification into high-risk, medium-risk, and low-risk groups. Compared with all competing methods, the overall accuracy of survival risk stratification into high-risk, medium-risk, and low-risk groups is 82.3% by the proposed model. The reason for such improvement is basically derived by employing a cross-attention mechanism along with Elastic Weight Consolidation methods, allowing the model to adapt to new patient data without catastrophic forgetting; hence, it showed strong accuracy in stratifying patients to appropriate risk categories.

**Table 6:** Model interpretability (average SHAP feature importance).

Method	Tumor size	EGFR mutation	Age	Comorbidities	Overall interpretability (SHAP score)
Proposed	0.312	0.278	0.193	0.161	0.736
Method^2^	0.281	0.252	0.176	0.143	0.682
Method^14^	0.269	0.245	0.163	0.137	0.664
Method^12^	0.256	0.232	0.154	0.128	0.634

Interpretability using SHAP feature importance scores of the models. The overall interpretability was highest for the proposed model, as reflected by its SHAP score of 0.736, which conveys that there is better explanation about its decisions. The model with the proposal gave more importance to tumor size and EGFR mutations in lung cancer predictions; therefore, clinicians can have more confidence in the decision-making process of the model. This interpretability, coupled with high predictive accuracy, makes the proposed model highly suitable for clinical applications in essence. We then discuss one practical use case for the proposed model, which shall help readers to further understand the entire process (Table 6).

### Practical use case scenario analysis

In the proposed work, a multi-model deep learning framework was compared using different processing steps: U-Net for tumor segmentation, followed by feature-level fusion and survival prediction using DeepSurv, with continuous adaptation of the model using EWC. Further, Bayesian Optimization was adopted for hyperparameter tuning, CAN to improve multi-modality data samples integration, and SHAP towards model interpretability. Testing of each process was performed with input samples comprising a patient’s CT scan, histopathology images, genomic data, and clinical information, such as age, co-morbidities, and survival outcomes. Sample P001 through P005 belongs to lung cancer patients with different tumoral characteristics, genomic profiles, and clinical histories, representative of the varied cases from TCGA and TCIA. P001 was a 65-year-old patient diagnosed with adenocarcinoma, presenting a tumor of 45.3 mm in diameter with EGFR mutations and with diabetes as comorbidity - a high-risk patient. P002 was a 58-year-old squamous cell carcinoma patient, presenting a tumor of 38.1 mm in diameter without major comorbidities, belonging to the medium risk concerning survival. P003 is also an adenocarcinoma, 72-year-old male patient, but his tumor size was 52.7 mm, with KRAS mutations. His co-morbidity of hypertension made him fall under the high-risk group. P004 is a 49-year-old man with large cell carcinoma and a tumor of 28.6 mm. No significant co-morbidities were identified in this patient. This patient was classified as low risk because his clinical and genomic markers were relatively better. P005 is finally 74 years old, presenting a 60.4 mm tumor of EGFR mutations with cardiovascular disease, hence at high risk to survive adenocarcinoma. Such cases were used in the evaluation of the multi-model deep learning framework across different subtypes of lung cancer and their associated survival predictions. The output values at every stage of the model pipelines are given in the following tables. These tables elaborate on tumor features extracted, feature-level fusion output, survival predictions, optimized hyperparameters among other results. It follows that the results show the efficiency of the model in the discriminative classification of subtypes of lung cancers and the prediction of survival outcomes based on an adaptability feature to new patient data through continuous learning mechanisms.

The integrated framework’s clinical decision-making interpretability improves using SHAP-based feature attributions. Clinicians can use model results in diagnosis and therapy by quantifying each variable’s risk stratification and categorization contribution. Radiographic signs like lesion compactness and spiculation and genetic factors like EGFR and KRAS mutation status dominated SHAP survival risk calculations. These linkages improve model transparency and clinical dependability by providing interpretable oncological evidence.

The framework’s interpretability helps oncologists evaluate treatment plans. High SHAP scores for EGFR mutations and tumor heterogeneity indicate aggressive biology. Even with poor radiography, such outputs can help oncologists recommend early targeted or combination therapy. Conservative care or conventional chemotherapy, an evidence-based precision strategy, may work for patients with lower genetic marker SHAP contributions but higher imaging stability. Interpretability results help radiologists reevaluate unclear imaging. A low-risk lesion may have disproportionately large SHAP contributions from hereditary variables, causing physicians to reevaluate imaging interpretation and explore confirmatory molecular testing. Visual-molecular interpretability synergy reduces heterogeneous cancer diagnoses with contradictory inputs.

Quantifying SHAP attributions improves prognosis beyond categorization. By correlating high cumulative SHAP scores to lower expected survival time, clinicians might identify patients who need close monitoring or early intervention. The explainable association between model predictions and patient-level variables makes computational outputs relevant for data-driven, individualized decision-making. The model becomes prediction system-assisted therapeutic reasoning with SHAP-based interpretability. Model outputs are transparent and contextual, allowing practitioners to compare computational assumptions to clinical experience. Trust, teamwork between radiologists, pathologists, and oncologists, and customized cancer care result from this.

**Table 7:** U-Net with iterative feature extraction (segmented features).

Patient ID	Tumor size (mm)	Tumor shape irregularity	Dice coefficient (CT)	Dice coefficient (histopathology)	Iteration count
P001	45.3	0.72	0.945	0.918	5
P002	38.1	0.68	0.928	0.904	4
P003	52.7	0.77	0.952	0.929	6
P004	28.6	0.65	0.912	0.890	4
P005	60.4	0.81	0.963	0.940	6

The results from U-Net with Iterative Feature Extraction give evidence of the model’s precision in segmenting tumor regions from CT and histopathology images and samples. This iterative model allowed an increase in segmentation accuracy across successive iterations, as found with larger and more irregular tumors. The Dice Coefficient is a measure of segmentation overlap against ground truth; thus, ranging between 0.890 and 0.963, it assuredly will bring out an excellence in segmentation process (Table 7).

**Table 8:** Feature-level fusion using CNN for images and MLP for genomic data.

Patient ID	Spatial features (CNN)	Genomic features (MLP)	Fused feature vector (dimensionality)
P001	[0.56, 0.44, 0.78]	[0.61, 0.75, 0.69]	[1.17, 1.19, 1.47]
P002	[0.63, 0.52, 0.74]	[0.58, 0.68, 0.71]	[1.21, 1.20, 1.45]
P003	[0.68, 0.55, 0.81]	[0.66, 0.79, 0.73]	[1.34, 1.34, 1.54]
P004	[0.52, 0.47, 0.69]	[0.57, 0.71, 0.64]	[1.09, 1.18, 1.33]
P005	[0.75, 0.61, 0.88]	[0.72, 0.81, 0.77]	[1.47, 1.42, 1.65]

In Table 8, the CNN-extracted spatial features from CT and histopathology images are fused at the feature level with the MLP-derived biological features from the genomic data samples. This mechanism of feature-level fusion combines such high-dimensional features, resulting in a much richer representation of the patient-specific tumor characteristics. Thus, the fused feature vector depicts synergy between the spatial and biological information, which is essential to the process of lung cancer subtype classification.

**Table 9:** DeepSurv survival prediction (hazard ratio).

Patient ID	Age	Tumor features	Genomic features	Comorbidities	Predicted hazard ratio	Risk group
P001	65	[45.3, 0.72]	[0.61, 0.75]	Diabetes	1.43	High
P002	58	[38.1, 0.68]	[0.58, 0.68]	None	1.08	Medium
P003	72	[52.7, 0.77]	[0.66, 0.79]	Hypertension	1.55	High
P004	49	[28.6, 0.65]	[0.57, 0.71]	None	0.92	Low
P005	74	[60.4, 0.81]	[0.72, 0.81]	Cardiovascular	1.67	High

Table 9 presents the survival predictions from the DeepSurv model. The hazard ratio was calculated by using the clinical data of patient age and comorbidities, image-derived tumor features, and genomic data samples. Because the predicted hazard ratios, the model has thus placed patients into three groups: high, medium, and low risk. As an example, the patient P005, having a large tumor size and comorbidity due to cardiovascular disease, falls into the high-risk group because of a hazard ratio set to 1.67 levels.

**Table 10:** Elastic weight consolidation (EWC) update results.

Patient ID	Old hazard ratio	New hazard ratio (after incremental update)	Importance of old parameters (EWC)	Change in model performance (%)
P001	1.43	1.42	0.87	+ 1.2
P002	1.08	1.09	0.82	+ 0.9
P003	1.55	1.53	0.91	+ 1.4
P004	0.92	0.93	0.85	+ 0.7
P005	1.67	1.66	0.93	+ 1.5

Table 10 underlines the result of Elastic Weight Consolidation that allowed the model to adapt incrementally without experiencing catastrophic forgetting. The updated hazard ratios remained close to the original predictions, while the model performance slightly increased by about 1–1.5.5%. This EWC mechanism ensures that parameters important for previous predictions remain preserved during learning new data samples.

**Table 11:** Bayesian optimization hyperparameter tuning.

Parameter name	Initial value	Optimal value (after Bayesian optimization)	Improvement in convergence speed (%)	Reduction in loss (%)
Learning rate	0.001	0.0005	12.5	8.4
Dropout rate	0.3	0.2	9.7	6.1
CNN layers	4	5	15.0	7.8
MLP layers	3	4	10.2	5.5

Table 11 presents the results of Bayesian Optimization applied to the model hyperparameters. It found the optimum in terms of learning rate, dropout rate, and number of layers for CNN and MLP architectures that provided taster convergence speeds along with lower loss but higher overall model performances.

**Table 12:** Cross-attention network (CAN) feature weights.

Patient ID	Imaging attention weight	Genomic attention weight	Combined attention weight
P001	0.61	0.39	0.53
P002	0.55	0.45	0.50
P003	0.64	0.36	0.55
P004	0.49	0.51	0.50
P005	0.68	0.32	0.54

In Table 12, the Cross-Attention Network estimated dynamic weights for the features from both imaging and genomic data samples. In the case of patient P005, high attention weight 0.68 was assigned to imaging data, representing the importance of spatial tumor characteristics in this specific case. The combined attention weight was used for final prediction by each model to focus on the most relevant features from each of the patients.

**Table 13:** Shapley additive explanations (SHAP) for model interpretability.

Patient id	Tumor size	EGFR mutation	Age	Overall SHAP score
P001	0.35	0.28	0.17	0.80
P002	0.31	0.26	0.18	0.75
P003	0.37	0.31	0.21	0.89
P004	0.28	0.22	0.15	0.65
P005	0.42	0.33	0.20	0.95

SHAP values to explain the model outcome. This plots the contribution of every feature-tumor size, EGFR mutation, and age-toward the final prediction. In the case of a patient, P005, tumor size is the most influencing feature among all the features, followed by the EGFR mutation with a cumulative SHAP score of 0.95. High interpretability on this level can provide greater transparency into how the model has made its decision, hence building clinical trust (Table 13).

**Table 14:** Final outputs for subtype prediction and survival risk stratification.

Patient ID	Predicted subtype	Predicted survival risk group	SHAP explanation score
P001	Adenocarcinoma	High	0.80
P002	Squamous cell	Medium	0.75
P003	Adenocarcinoma	High	0.89
P004	Large cell	Low	0.65
P005	Adenocarcinoma	High	0.95

The final outputs of the model for lung cancer subtype classification and survival risk stratification are summarized in Table 14. Indeed, the model predicted the correct subtypes of lung cancer and appropriate stratification of patients according to their survival risk. A 6% misclassification of large cell carcinoma as squamous cell carcinoma was the most common. CT and histopathological imaging shared physical characteristics, especially when tumor borders were irregular defined, causing misinterpretation. Genomic data in multimodal fusion phases revealed unique mutation trends in these subtypes, minimizing misdiagnosis. Unique EGFR mutation profiles and consistent glandular imaging patterns classified adenocarcinomas. Imaging blurs subtype boundaries, but genetic cues improve classification reliability and clinical interpretability, making multimodal integration essential. All these predictions were supported by SHAP explanation scores for each patient, thus further asserting that those predictions were indeed good accurate and reliable in the process.

## Conclusion and future scopes

Finally, the proposed multimodal deep learning approach with lung cancer subtype classification and survival prediction showed best performance in several tasks: imaging, genomic, and clinical data samples. As a result, the iterative feature extraction architecture of U-Net is able to yield an average Dice coefficient of 0.926 for segmenting tumors that are remarkably improved compared to current methods^2,12,14^ with Dice coefficients of 0.891, 0.874, and 0.852 respectively. Due to the feature-level fusion, it combined CNNs for image processing with MLPs for genomic data, allowing it to achieve a classification accuracy of 88.8%, outperforming other methods by about 4–7%. Further improvement was seen in AUC-ROC to 0.915 due to the cross-attention mechanism, enhancing the ability of correctly distinguishing lung cancer subtypes such as adenocarcinoma, squamous cell carcinoma, and large cell carcinoma. The C-index for the DeepSurv model with EWC, as applied to survival time prediction, was 0.795, illustrating its robustness in the survival time prediction and risk stratification of the patients. Comparatively, the results from methods^2,12,14^ were 0.768, 0.752, and 0.737, respectively. EWC’s incremental learning capability ensured that new patient data was able to be integrated without loss of performance, enhancing the prediction accuracy by about 10%. Similarly, SHAP provided high interpretability, given that feature importance scores-including tumor size SHAP score of 0.42-offered insight into the decision-making process and, thus, enhanced clinical trust in the model outputs. These results are testimony that the framework proposed in this paper yields highly accurate, interpretable, and adaptive results regarding the diagnosis and prognosis of lung cancer sets.

### Future scope

This work, despite the remarkable advances shown in it, merits further consideration of several areas of interest with a view to improving the framework’s clinical applicability and scalability. Further development should be directed towards the extension of this dataset through further diversification and enlargement of patient cohorts, considering even rarer subtypes of lung cancer and various stages of disease progress with a view towards generalizability across larger populations. It can be extended further to even more real-time multimodal data streams, including longitudinal imaging and dynamic genomic changes due to treatment, which would confer even greater strengths in the prediction of the response of treatments and updating survival predictions over temporal instance sets. Other areas of improvement are in computational efficiency optimization within the current framework, particularly for iterative U-Net segmentation and Bayesian optimization processes to enable real-world application on resource-constrained clinical deployment platforms. Therefore, further investigation of sophisticated attention mechanisms, including multi-head self-attention and transformer architectures, could further optimize the integration of imaging and genomic data and achieve classification accuracy above 90% while enhancing the robustness of survival predictions. This might be developed into more interpretable models using advanced explainability tools, such as counterfactual explanations or causal inference models, to provide actionable insights for clinicians in personalized treatment strategies. All in all, the developed framework can be expanded in the future toward treatment recommendation systems that integrate predicted subtypes and survival risks for coming up with optimal therapeutic interventions, therefore making the support holistic for precision oncology sets.

## Data Availability

The datasets used and/or analysed during the current study available from the corresponding author on reasonable request.

## Appendix

**Table 15:** Empirical review of existing methods.

Reference	Method used	Findings	Results	Limitations
^1^	Transformer-Based Approach	Electronic claims data used for lung cancer prediction	Achieved high prediction accuracy	Limited generalizability to non-claims datasets
^15^	Electrical Properties Analysis	Lung nodule properties measured across frequencies	High sensitivity for squamous cell carcinoma detection	In vitro limitations; not applicable for real-time use
^2^	Respiratory Impedance Parameterization	Impedance-based lung cancer diagnosis	Accurate modeling of respiratory biomechanics	Limited to specific obstructive disease conditions
^3^	Machine Learning with Cat Mouse Optimizer	CT imaging for lung cancer classification	Improved classification with feature extraction	Computational complexity in feature extraction
^16^	Spatial Pyramid Pooling with 3D CNN	Improved detection using 3D convolutions on low-dose CT scans	Enhanced detection accuracy by 15%	Requires large-scale 3D data, increasing computational costs
^17^	Deep Learning with Multi-Omics Data	Multi-omics data improves lung cancer classification	High accuracy for subtype prediction	Limited data availability for rare subtypes
^6^	Fully Automated Screening System	End-to-end automation of lung cancer screening	High accuracy with automated segmentation	Generalization issues for unseen imaging modalities
^14^	AI-Driven Drug Effectiveness-Cost Analysis	Synthetic biology models for drug cost analysis	Optimized cost-effectiveness in treatment	Focused on non-small cell lung cancer only
^18^	RetinaNet for Lung Cancer Detection	RetinaNet used for multi-scale feature fusion in early detection	Increased early detection rates	Sensitive to image resolution variability
^19^	AI for Histological Image Analysis	Hybrid AI models for histopathological analysis	High accuracy in early diagnosis	Limited to histological data; lacks multimodal integration
^20^	Radiomics-Based NSCLC Characterization	Automated radiomics pipeline for NSCLC	Accurate characterization with PET/CT	Dependent on high-quality radiomic data
^21^	Tuna Swarm Algorithm with Deep Learning	Algorithm used for colon and lung cancer detection	Competitive detection rates compared to standard methods	Requires extensive training data for swarm optimization
^5^	Multimodality 3D Detection in PET/CT	3D convolutional networks improve detection of lung cancer	Significant improvements in detection rates	High data acquisition costs with PET/CT
^22^	Modified U-Net with SVM	Lobe segmentation and nodule detection using U-Net and SVM	Achieved competitive classification accuracy	Limited scalability for larger datasets
^12^	CNN for Tumor Detection in CT Images	CNN-based tumor detection using CT scan data	Improved tumor detection with active counter	High computational demand for real-time analysis
^23^	EGFR Mutation Drug Resistance Modeling	Computational methods for predicting drug resistance	Enhanced drug design for lung cancer treatments	Limited to NSCLC with EGFR mutations
^24^	Antenna-Based Biomedical Imaging	Use of antennas for lung cancer detection	High accuracy with minimal invasiveness	Requires further validation for clinical use
^25^	Bioinformatics and Random Forest	Random forest used for squamous cell lung cancer prognosis	Improved survival analysis using gene signatures	Limited interpretability of random forest models
^26^	Transfer Learning with Class Selective Processing	Transfer learning for lung and colon histopathology	Increased accuracy using selective processing	Limited by availability of labeled histopathological data
^11^	Rho-GDI Pathway Network Modeling	Integrative modeling of NSCLC progression pathways	Highlighted Rho-GDI signaling importance in cancer	Limited to specific pathway analyses
^27^	Water Strider Algorithm for Cancer Detection	Histopathology-based detection using deep learning and swarm algorithms	Improved cancer detection	Computationally expensive for real-time application
^4^	Image-Genomics Data Fusion	Hybrid deep networks for subtype diagnosis	Effective in fusing imaging and genomic data	Requires significant computational resources
^28^	Bidirectional Association Discovery	Precise lung cancer biomarker identification using clustering	High accuracy in genome sequence analysis	Limited to gene expression datasets
^29^	Modality-Specific PET-CT Segmentation	Segmentation using a modality-specific network for lung tumors	High segmentation accuracy	Data availability is restricted to PET-CT imaging
^30^	RadioPathomics for Adaptive Radiotherapy	Multimodal learning for NSCLC	Improved radiotherapy outcomes using pathomics	Requires complex multimodal data integration
^31^	Fuzzy Attention Neural Networks	Tackles discontinuity in airway segmentation using fuzzy logic	Improved segmentation for pulmonary fibrosis	Limited application to airway segmentation problems
^32^	Mask-Guided Deep Learning Framework	Distant metastasis prediction using deep learning	Accurate prediction of metastasis	Complex models increase computational burden
^33^	Support Vector Machine for Tumor Detection	Lung tumor detection in mice using SVM and LIBS	High tumor detection rates	Limited to animal models; requires validation in humans
^34^	Global Context Attention with CNN	Histopathological image classification using global context attention	Improved classification accuracy for lung and colon cancer	Requires large labeled datasets
^9^	Ambiguous Label Learning for Nodule Prediction	Learning from ambiguous labels for malignancy prediction	Enhanced nodule malignancy prediction	Dependent on accurate ambiguous labeling strategies
^35^	Reconstruction-Assisted Feature Encoding	Feature encoding for NSCLC subtype classification	Improved histologic subtype classification	Limited by histological reconstruction capabilities
^36^	Electrical Impedance Spectroscopy	Differentiating between healthy and neoplastic lung tissues	High sensitivity using minimally invasive methods	Limited to specific tumor types
^37^	Co-Modules for Cancer Subgroup Identification	Identifying lung cancer subgroups using co-modules	Accurate patient subgroup identification	Limited to driver mutation profiles
^38^	Rough Set Theory for Gene Triplets	Mining genetic interaction triplets for lung adenocarcinoma	Identified key genetic interactions	Focused on adenocarcinoma; lacks generalization
^8^	Dual-Branch Framework for Lung Nodule Segmentation	Precise segmentation of lung nodules using feature fusion	Achieved high segmentation accuracy	Requires prior knowledge for accurate segmentation
^39^	Textural Analysis of PET Imaging	Textural analysis improves NSCLC detection in PET imaging	Enhanced detection using 3D analysis	Limited to PET imaging data only
^40^	Multiscale Aggregation Network	Lung nodule detection using multiscale CNN	Accurate detection with self-calibrating convolution	Requires high computational resources for scaling
^41^	Electrochemical Aptasensor for Biomarker Detection	Dual biomarker detection using aptasensors	Highly sensitive for EGFR and NSE detection	Requires advanced lab infrastructure for implementation
^42^	3D CNN with Optical Flow	Pulmonary nodule detection using optical flow in CT scans	Increased detection rates	Requires large-scale 3D datasets for training
^43^	DNN-Assisted Terahertz Sensors	Detection of lung cancer biomarkers using DNN and terahertz sensors	High sensitivity in miRNA detection	Limited by high-cost sensor technologies

Indeed, Table 15 reflects an active field with rapid technological advances pressing hard to meet the delights of precision medicine. Of course, just recently, outstanding achievements in areas such as early detection, diagnosis, and survival prediction have indeed been attained by using state-of-the-art advanced machine learning techniques, but yet there remains the trinity of challenges in data availability, computation scalability, and model interpretability. In fact, now is a point in time when the area dynamically develops, and the integration of multi-modal data, domain knowledge, and interpretability mechanisms is a path of crucial importance while developing new generations of AI-based lung cancer management tools. Moreover, as these models are further refined and validated through clinical trials, their potential clinical usefulness could be significantly enhanced and might even change the face of lung cancer diagnosis and treatment.
